# Evaluation of Bioelectrical Impedance Analysis for Identifying Overweight Individuals at Increased Cardiometabolic Risk: A Cross-Sectional Study

**DOI:** 10.1371/journal.pone.0106134

**Published:** 2014-09-22

**Authors:** Maxine J. E. Lamb, Christopher D. Byrne, James F. Wilson, Sarah H. Wild

**Affiliations:** 1 Centre for Population Health Sciences, University of Edinburgh, Edinburgh, United Kingdom; 2 Nutrition and Metabolism, Faculty of Medicine, University of Southampton, Southampton, United Kingdom; 3 Southampton National Institute for Health Research Biomedical Research Centre, University Hospital Southampton, Southampton, United Kingdom; 4 MRC Institute of Genetics and Molecular Medicine, University of Edinburgh, Western General Hospital, Edinburgh, United Kingdom; Bielefeld Evangelical Hospital, Germany

## Abstract

**Objective:**

To investigate whether bioelectrical impedance analysis could be used to identify overweight individuals at increased cardiometabolic risk, defined as the presence of metabolic syndrome and/or diabetes.

**Design and Methods:**

Cross-sectional study of a Scottish population including 1210 women and 788 men. The diagnostic performance of thresholds of percentage body fat measured by bioelectrical impedance analysis to identify people at increased cardiometabolic risk was assessed using receiver-operating characteristic curves. Odds ratios for increased cardiometabolic risk in body mass index categories associated with values above compared to below sex-specific percentage body fat thresholds with optimal diagnostic performance were calculated using multivariable logistic regression analyses. The validity of bioelectrical impedance analysis to measure percentage body fat in this population was tested by examining agreement between bioelectrical impedance analysis and dual-energy X-ray absorptiometry in a subgroup of individuals.

**Results:**

Participants were aged 16-91 years and the optimal bioelectrical impedance analysis cut-points for percentage body fat for identifying people at increased cardiometabolic risk were 25.9% for men and 37.1% for women. Stratifying by these percentage body fat cut-points, the prevalence of increased cardiometabolic risk was 48% and 38% above the threshold and 24% and 19% below these thresholds for men and women, respectively. By comparison, stratifying by percentage body fat category had little impact on identifying increased cardiometabolic risk in normal weight and obese individuals. Fully adjusted odds ratios of being at increased cardiometabolic risk among overweight people with percentage body fat ≥25.9/37.1% compared with percentage body fat <25.9/37.1% as a reference were 1.93 (95% confidence interval: 1.20–3.10) for men and 1.79 (1.10–2.92) for women.

**Conclusion:**

Percentage body fat measured using bioelectrical impedance analysis above a sex-specific threshold could be used in overweight people to identify individuals at increased cardiometabolic risk, who could benefit from risk factor management.

## Introduction

Thresholds of body mass index (BMI) are used worldwide to identify people who are normal weight, overweight or obese. Although it is recognised that obesity, defined by a BMI ≥30 kg/m^2^ in Europeans, is a cause of impaired health and disease [1], BMI does not provide information on fat mass or percentage, or fat distribution which are more strongly related to cardiometabolic risk than BMI [2].

As BMI measurement is a poor predictor of cardiometabolic risk, other simple alternative anthropometric measures have been tested, and waist circumference (WC), either alone or in combination with other anthropometric measurements is considered more useful for identifying individuals at increased cardiometabolic risk [3], [4] than BMI [5]. However, WC has not been widely adopted in clinical practice both because it is inconvenient for patients and health professionals, and the variability introduced by different measurement sites and imprecision of measurement leads to poor reproducibility [6]. Poor reproducibility is particularly important if dichotomous cut-points are used to define at risk groups for further assessment [7], [8].

The limitations of BMI and WC, coupled with a need for quick and accurate measurements in clinical practice, have led to renewed interest in alternative measurements of body composition, such as bioelectrical impedance analysis (BIA). BIA works by determining the electrical impedance of a small constant alternating current passing through the body [9] and can be measured by a variety of devices. Population-specific models have been created to use an individual's impedance value to estimate percentage body fat (%BF) [10], [11]. Unlike for BMI, %BF thresholds have not been ascertained that identify high risk subgroups. Since BIA is a simple, non-invasive, inexpensive and portable body composition method, it may be a useful screening technique when used in combination with BMI to identify individuals who are at increased cardiometabolic risk and need further investigation. Our aim was therefore to test whether %BF measured by BIA would provide a useful addition to measurement of BMI to identify individuals who are at increased cardiometabolic risk, defined by the presence of the metabolic syndrome (using international consensus criteria [12]) and/or diabetes. The validity of BIA to measure %BF was tested by examining agreement between BIA and dual-energy X-ray absorptiometry (DXA) considered to be the “gold standard” [13] in a subgroup of individuals.

## Materials and Methods

Between 2005 and 2011, individuals with at least one grandparent from the North Isles of Orkney were recruited to participate in the Orkney Complex Disease Study (ORCADES), a genetic epidemiology study [14]. Ethical approval was given by the North of Scotland multi-centre research ethics committee and all participants provided written informed consent. Recruitment was achieved through a variety of measures, including requests for volunteers through advertisements in the local newspaper and on the local radio; posters in public places; talks to organisations and community leaders; and requesting contact information of eligible individuals from volunteers. Weight and height were measured with footwear removed and in light clothing. Waist measurement was performed using a rigid tape measure at the midpoint between the lower rib and iliac crests. BIA was conducted using a foot-to-foot Tanita UM-014 bioimpedance analyser (Tokyo, Japan) where the individual stood barefoot on metal plates in light clothing and %BF was recorded. DXA measurements were taken using a Hologic QDR4500 scanner (Bedford, MA). The scan was completed with the individual in light clothing and in a supine position. Blood samples were collected after an overnight fast.

Increased cardiometabolic risk status was defined as having one or both of diabetes and metabolic syndrome (see below for definition). Diabetes was defined as the presence of self-reported diabetes and/or HbA1c levels ≥6.5%. The metabolic syndrome was defined using the International Diabetes Federation consensus definition criteria for systolic and diastolic blood pressure (≥130 mmHg and ≥85 mmHg, respectively), high density lipoprotein (HDL; ≤1 mmol/l in men and ≤1.3 mmol/l in women), glucose (≥5.6 mmol/l), triglycerides (≥1.7 mmol/l) and WC (≥94 cm in men and ≥80 cm in women) [12]. People treated with hypertension medication were assumed to meet the blood pressure criteria. Lipid lowering drugs that may influence HDL or triglyceride levels were not used widely in this population therefore their use was not considered in the analysis. Individuals above the thresholds for three or more of the above factors were considered to have the metabolic syndrome.

### Data analysis

Demographic and clinical variables that were not normally distributed were reported as median (interquartile range); otherwise variables were reported as mean ± standard deviation. Receiver-operating characteristic (ROC) curves were used to generate an optimum sex-specific %BF threshold. ROC curves were also generated for BMI to enable comparison of the area under the curve (AUC). The prevalence of increased cardiometabolic risk in this population with %BF below and equal to or above the ROC-identified thresholds, stratified by BMI in conventional categories (<25, 25−<30 and ≥30 kg/m^2^), was calculated. Multivariable logistic regression was used to calculate odds ratios and 95% confidence intervals (ORs 95%CIs) for increased cardiometabolic risk for %BF and BMI categories, adjusting for age and smoking status. Due to the small number of individuals with %BF <25.9/37.1% and BMI ≥30 kg/m^2^, and %BF ≥25.9/37.1% and BMI <25 kg/m^2^ these groups were not included in the analysis. Multivariable logistic regression was also used to estimate the OR for increased cardiometabolic risk for the high compared to low %BF groups among people with BMI 25−<30 kg/m^2^. All analyses were stratified by sex.

A subgroup of individuals with DXA measurements were used to examine the agreement between BIA and DXA in this population, stratified by sex. BIA and DXA measurements were not always conducted on the same day therefore these analyses were limited to individuals whose weight was within two kilograms to minimise variation caused by changes in body mass. Demographic characteristics were compared between the subgroup and the rest of the population using two-sample t-tests for normally distributed characteristics and Mann-Whitney *U*-test for non-normally distributed data. ROC analysis was used to establish appropriate %BF thresholds for both BIA and DXA. Difference in mean %BF was calculated as %BF_BIA_-%BF_DXA_. Data were normally distributed so paired t-tests were conducted and p-values reported. P-value <0.05 was considered significant. Limits of agreement were calculated as mean difference ± two standard deviations and Bland-Altman plots of individuals' mean %BF against the difference of the two %BF measurements were drawn [15].

All analysis was conducted using Stata v11 (College Station, Texas).

## Results

Demographic and clinical information for the study population of 1998 individuals whose age ranged from 16–91 years is shown in Table 1. Almost one-third of the population had increased cardiometabolic risk with higher prevalence among men than women. BMI and %BF were strongly correlated (R = 0.81 in men, 0.84 in women). AUCs for being at increased cardiometabolic risk were very similar for BMI and %BF in men (BMI: 0.76 (95% CI: 0.73 to 0.79), %BF: 0.77 (0.73 to 0.80)) and women (BMI: 0.77 (0.75 to 0.80), %BF: 0.77 (0.74 to 0.80)).

**Table 1:** Characteristics of study population, stratified by sex.

	Men	Women
**N (%)**	788 (39.44)	1210 (60.56)
**Age (years)**	54.29±14.81	53.45±15.11
**Height (cm)**	175.17±6.45	161.22±6.04
**Weight (kg)**	85.92±12.58	71.40±14.40
**BMI (kg/m^2^)**	27.59 (5.03)	26.41 (6.61)
**%BF (%)**	26.53±6.72	36.55±7.33
**WC (cm)**	98.62±11.86	89.22±13.56
**SBP (mmHg)**	134.33 (16.89)	127.11 (19.63)
**DBP (mmHg)**	77.35±9.59	74.25±9.21
**Hypertension medication (%)**	166 (21.07)	237 (19.59)
**Glucose (mmol/l)**	5.3 (0.7)	5.1 (0.6)
**Triglycerides (mmol/l)**	1 (0.8)	0.9 (0.6)
**HDL (mmol/l)**	1.32±0.37	1.61±0.43
**Increased cardiometabolic risk (%):**	293 (37.37)	322 (26.95)
**Type 2 diabetes (%)**	49 (6.22)	51 (4.22)
**Metabolic Syndrome (%)**	282 (35.97)	307 (25.69)
**Current smokers (%)**	65 (8.40)	97 (8.12)

Values are reported as mean ± standard deviation, except BMI, glucose and triglycerides which are reported as median (interquartile range). Increased cardiometabolic risk is defined as having diabetes and/or the metabolic syndrome. Proportions are reported for hypertension medication, increased metabolic risk, type 2 diabetes, metabolic syndrome and smoking status as n (%). %BF = percentage body fat; BMI = body mass index; DBP = diastolic blood pressure; HDL = high density lipoprotein; SBP = systolic blood pressure; WC = waist circumference.

Optimum thresholds for identifying individuals at increased cardiometabolic risk from %BF were 25.9% for men and 37.1% for women. Approximately half (51%) of individuals at increased cardiometabolic risk had a BMI <30 kg/m^2^. The majority of these individuals had a BMI in the ‘overweight’ category (25−<30 kg/m^2^). In individuals with BMI 25−<30 kg/m^2^, the prevalence of increased cardiometabolic risk in those with %BF above the threshold was double the prevalence in those with %BF below the threshold (Table 2).

**Table 2:** Number of individuals and prevalence of being at increased cardiometabolic risk in individuals whose %BF is above and below the sex-specific threshold, stratified by BMI and sex.

	Increased cardiometabolic risk			
	Men		Women	
	%BF<25.9%	%BF≥25.9%	%BF<37.1%	%BF≥37.1%
**BMI <25 kg/m^2^**	11 (6.96%)	3 (50.00%)	22 (5.34%)	4 (17.39%)
**BMI 25−<30 kg/m^2^**	53 (23.56%)	85 (48.02%)	34 (18.89%)	101 (37.97%)
**BMI** **≥30 kg/m^2^**	4 (66.67%)	137 (64.32%)	2 (33.33%)	159 (51.62%)

%BF = percentage body fat; BMI = body mass index.

Both crude and adjusted odds of increased cardiometabolic risk were significantly higher for all groups when comparing with %BF <25.9/37.1% and BMI <25 kg/m^2^ as the reference group (Table 3). As expected, the highest odds were found when individuals with %BF ≥25.9/37.1% and BMI ≥30 kg/m^2^ were compared with the reference group. Odds of increased cardiometabolic risk were higher when comparing individuals who were above the %BF threshold with BMI 25−<30 kg/m^2^ with the reference group than when comparing below the %BF threshold and BMI 25−<30 kg/m^2^ with the reference group. Similar relationships were found in both men and women.

**Table 3:** Crude and adjusted odds ratios (OR), with 95% confidence intervals, of being at increased cardiometabolic risk with %BF above and below sex-specific thresholds (25.9% for men, 37.1% for women) and BMI <25 kg/m^2^, 25−<30 kg/m^2^ and ≥30 kg/m^2^ compared with %BF<25.9/37.1% and BMI <25 kg/m^2^.

	Men			Women		
	N	Crude OR	Adjusted OR*	N	Crude OR	Adjusted OR*
**%BF <25.9/37.1% & BMI <25 kg/m^2^**	160	1.00 (ref)	1.00 (ref)	422	1.00 (ref)	1.00 (ref)
**%BF <25.9/37.1% & BMI 25−<30 kg/m^2^**	225	4.12 (2.07–8.17)	4.44 (2.07–9.51)	181	4.13 (2.34–7.30)	3.61 (1.99–6.56)
**%BF ≥25.9/37.1% & BMI 25−<30 kg/m^2^**	179	12.49 (6.33–24.65)	9.75 (4.51–21.05)	269	10.85 (6.61–17.81)	6.47 (3.84–10.88)
**%BF ≥25.9/37.1% & BMI** **≥30 kg/m^2^**	213	24.41 (12.44–47.91)	20.18 (9.53–42.76)	309	18.92 (11.66–30.70)	15.82 (9.63–25.98)

%BF  =  percentage body fat; BMI  =  body mass index.

*adjusted for age and smoking status.

In the overweight group (BMI 25−<30 kg/m^2^), the adjusted ORs for increased cardiometabolic risk for the group with %BF ≥25.9/37.1% were 1.93 (1.20 to 3.10) for men and 1.79 (1.10 to 2.92) for women compared to the group with %BF <25.9/37.1%.

### Agreement of BIA and DXA

The subgroup included in the comparison of BIA with DXA data had a slightly lower proportion of men than in the rest of the population and mean age was slightly higher. In men, there were no significant differences between age, weight, BMI and %BF in the subgroup with DXA data available and the rest of the population. Conversely, all of the mean and median values were significantly different between the subgroup and the rest of the population in women. However, when the ROC analysis was conducted using only this subgroup of individuals, the %BF thresholds measured using BIA to identify individuals at increased cardiometabolic risk were very similar to those identified using the whole population (25.7% in men and 37.2% in women in the subgroup, compared with 25.9% in men and 37.1% in women in the whole population).

**Figure 1:**
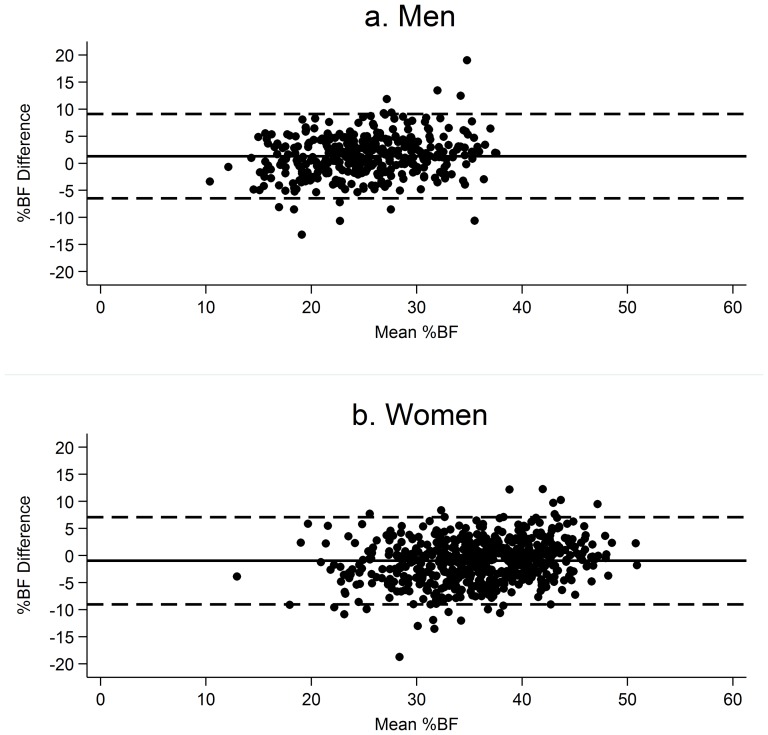
Bland-Altman plots showing the limits of agreement between percentage body fat measured by bioimpedance analysis (%BF_BIA_) and percentage body fat measured by dual-energy X-ray absorptiometry (%BF_DXA_) in men (a, n = 363) and women (b, n = 596). Mean difference is calculated by %BF_BIA_-%BF_DXA_ and the limits of agreement are calculated by mean difference ± two standard deviations. The solid line represents the mean difference and the dashed lines represent the limits of agreement.

BIA slightly overestimated %BF measured by DXA in men, and BIA underestimated %BF measured by DXA in women (Table 4). However, the limits of agreement were wide in both men and women. Figure 1 suggests that in both sexes BIA may overestimate %BF slightly more as mean %BF increases although there were no strong trends in either sex. Using ROC analysis, optimum thresholds were generated for use of DXA to identify those at increased cardiometabolic risk, in addition to those for BIA. The DXA threshold for men was 1.2% lower than that for BIA (24.7%), whereas the DXA threshold generated for women was 1.4% higher than that for BIA (38.6%). When comparing the efficacy of the two to similarly identify individuals, 107 individuals were categorised as above the threshold for BIA but not for DXA, and 69 individuals were above the DXA threshold but not the BIA threshold. This equates to 18.4% of the subgroup population in which classification by the two measures differed. In men and women the proportion of individuals the measures did not agree on were 20.7% and 17.0%, respectively, which suggests that the two measures show slightly better agreement when identifying women at increased cardiometabolic risk, than men.

**Table 4:** Agreement between BIA and DXA, stratified by sex.

	Men	Women
**N (%)**	363 (37.85)	596 (62.15)
**%BF_BIA_**	26.02±6.26	35.70±7.09
**%BF_DXA_**	24.71±5.46	36.64±6.27
**Mean difference (BIA-DXA)**	1.31 (0.90 to 1.71)*	−0.94 (−1.27 to −0.62)*
**Limits of agreement**	−6.55 to 9.17	−7.14 to 9.02

Mean values of %BF_BIA_ (%BF measured by BIA) and %BF_DXA_ (%BF measured by DXA), and the mean difference are reported as mean ± standard deviation. Limits of agreement were calculated as the mean difference ± 2 standard deviations. P-values were calculated by a paired t-test. %BF  =  percentage body fat; BIA  =  bioimpedance analysis; DXA  =  dual-energy X-ray absorptiometry. *P<0.001.

## Discussion

Our findings suggest that %BF measured using BIA above a sex-specific threshold could be used in the overweight group (defined by BMI 25−<30 kg/m^2^) to identify individuals at increased cardiometabolic risk. Sex-stratified AUCs for obesity defined by %BF, based on population specific cut-points (25.9% for men and 37.1% for women), and BMI defined as ≥30 kg/m^2^ were very similar. Prevalence of increased cardiometabolic risk was highest among people with BMI ≥30 kg/m^2^ and was lowest in the group with BMI <25 kg/m^2^. Prevalence of increased cardiometabolic risk among the BMI 25−<30 kg/m^2^ group was intermediate between the other BMI categories and application of %BF thresholds allowed stratification into lower and higher risk subgroups within this BMI category.

Previous studies have shown that a large proportion of people with normal or overweight BMI have metabolic abnormalities [16], [17]. WC is known to be valid either as an alternative to, or in addition to, BMI and it has been shown to correlate with cardiometabolic risk factors [3], [18]–[21]. The variability and lack of reproducibility of waist measurements [6], [20], [22], and the practical difficulties of measuring waist mean that it is not widely used in clinical practice.

Consistent with our findings, several previous studies have found there to be little or no difference in associations between BMI and %BF and various cardiometabolic risk factors [23]–[30]. Three of these studies were performed in Japanese populations and a fourth one, in an American population, suggested that there may be ethnic differences in the strength of association between BMI and %BF and cardiometabolic risk factors.

The use of %BF compared to BMI when assessing cardiometabolic risk factors has been investigated in four European studies [27]–[30]. Bosy-Westphal and colleagues reported AUCs for metabolic syndrome (defined as two or more of elevated blood pressure, triglycerides or glucose) among 335 Germans of 0.694 and 0.800 for BMI, and 0.691 and 0.698 for %BF (measured by air displacement plethysmography) for men and women, respectively [27]. Dervaux et al, in a French population of 649 subjects, found that an increase of one standard deviation of BMI showed a non-statistically significantly 20% higher increase in odds of metabolic syndrome than a one standard deviation increase of BIA-measured %BF [28]. Two studies use dichotomous thresholds for %BF to examine cardiometabolic risk. In a Spanish population (n = 6123), Gómez-Ambrosi and colleagues, used air displacement plethysmography to measure %BF and found there was a difference in risk factor patterns including C-reactive protein, total, low and high-density lipoprotein levels and systolic and diastolic BP between ‘non-obese by BMI and non-obese by %BF’ individuals and ‘non-obese by BMI but obese by %BF individuals’ [29]. Phillips and colleagues investigated differences between ‘obese by BMI and obese by %BF’ and ‘non-obese by %BF and obese by BMI’ in a European population with metabolic syndrome, measuring %BF by BIA [30]. They found there to be higher levels of several risk factors in the ‘obese by BMI and obese by %BF’ group compared to the ‘non-obese by BMI and obese by %BF’. These studies, in agreement with the present study, suggest that the complementary use of both BMI and measurement of %BF would improve detection of individuals at greater cardiometabolic risk over the sole use of BMI.

### Validity of BIA compared with DXA

In our study BIA was found to overestimate %BF compared to DXA in men and underestimate %BF in women, and 18.6% of the population would be classified into different risk groups by the two measures. Several studies used similar BIA equipment (foot-to-foot Tanita analysers) to this study in European populations with inconsistent results reporting no difference, overestimation and underestimation of %BF by BIA compared to DXA in various studies of men and women [31]–[35]. In previous studies, the limits of agreement range from approximately 10% to 16% [32]–[34] and were 15.7% in men and 16.2% in women in our study. The variation between studies may reflect differences in age distribution or the type of equipment used with the additional potential effect of changes over time between the two measurements in our study.

It appears that there are sex differences in the agreement of BIA and DXA in this population, therefore further investigation is warranted into the reliability of the use of BIA at an individual level. It may be sufficient to ensure that there are sex-specific thresholds appropriate to the type of body composition measure used – for example in this population, as BIA underestimates %BF in women, the DXA thresholds for increased risk are higher than the equivalent BIA threshold.

### Strengths and limitations

There are a number of strengths of this study. The large sample size enabled stratification by sex and use of both BIA and DXA measurements allowed their comparison in a subgroup of participants. The range of age and BMI of the participants is very broad although sensitivity analysis including only individuals with BMI 18–40 kg/m^2^ and aged 18–80 years gave similar results.

Mean BMI is very similar between the participants of the Scottish Health Survey, a nationally representative sample, and participants in this study [36]. However, as this study features an isolated, island population, it may be difficult to extrapolate these results to other populations, in which diet and lifestyles are different. Additionally, generalisation of body fat cut-points to a population which is not of white European origin is unlikely to be valid. Although the small numbers of people with BMI <25 kg/m^2^ and %BF equal to or above sex specific thresholds in this population suggest that BIA is likely to be of limited value in people with normal BMI further research in different populations is needed to confirm this finding.

BIA was selected as the primary exposure of interest for this analysis due to its increased feasibility and affordability for use in clinical practice and population studies, when compared with techniques such as DXA or air displacement plethysmography. However, BIA has many limitations. As %BF is calculated by a regression equation, the reliability of the estimate depends on the accuracy of the regression equation and therefore on similarity to the reference population. Foot-to-foot BIA may be further limited due to the single frequency impedance measure which may not be as precise as impedance values measured at multiple frequencies [37]. Additionally, as BIA calculates fat-free mass and fat mass by quantifying total body water, estimated %BF may be affected by food or fluid intake prior to measurement, hydration status, recent physical activity and certain medical conditions. Although a measure of body fat that is independent of total body mass might be preferable to derived measures such as BIA for assessing cardiometabolic risk such measures are not currently available for use in clinical practice.

It may also be appropriate to refine cut-points for different populations, for example to develop age and ethnic-specific cut-points. In order to provide simple, clinically useful measures we chose to define binary cut-points of body fat, similar to the binary categories used for BMI. The use of continuous measures in a computer based risk algorithm could potentially provide improved discrimination than that offered by definitions of threshold values although algorithms would still need to be calibrated appropriately for different populations. Some of the differences observed between BIA and DXA may have been due to the different timings of BIA and DXA measurements. We attempted to address this issue by limiting the comparisons to individuals whose body weight was within two kilograms at each measurement; however changes in %BF may have occurred over time.

Finally, it is important to acknowledge the value of identifying the presence of metabolic syndrome has been challenged and the debates over how it should be defined. However it is accepted that this cluster of risk factors are useful in identifying people at high risk of developing cardiovascular disease and that effective interventions are available to reduce risk [38].

## Conclusion

When planning the use of limited resources it is important that efficient approaches are used to identify individuals at increased cardiometabolic risk. Although WC adds valuable information to BMI its measurement has not been widely incorporated into clinical practice. In our study, %BF measured by BIA identified individuals at increased cardiometabolic risk among people with BMI 25−<30 kg/m^2^ suggesting that BIA could provide a useful screening tool in clinical practice to identify people of European ancestry in this BMI category who should have their risk factors measured and managed. Further research into the value of %BF measured by BIA as a marker of metabolic health would be beneficial.
